# Evaluation of winter pressures on general practice in Manchester: a cross-sectional analysis of nine GP practices

**DOI:** 10.3399/bjgpopen20X101138

**Published:** 2021-01-13

**Authors:** Sinead Millwood, Peter Tomlinson, Jon Hopwood

**Affiliations:** 1 GPST3, Ashville Surgery Manchester, Manchester, UK; 2 Data Analyst, Manchester Primary Care Partnership Limited, Manchester, UK; 3 GP Partner, Ashville Surgery Manchester, Manchester, UK; 4 Director, Primary Care Manchester Limited, Victoria Mill, Manchester, UK; 5 Clinical Director, West Central Manchester Primary Care Network, Manchester, UK

**Keywords:** primary health care, general practice, winter pressures, seasons, resilience, improving access, workload

## Abstract

**Background:**

The Nuffield Trust’s report on NHS winter pressures highlights a lack of data for primary care, with a consequential focus on secondary care. An increase in data is required on the scale of the winter demand on primary care, so the need for investment in this area can be clearly seen.

**Aim:**

To quantify seasonal variation in workload in primary and secondary care.

**Design & setting:**

Analysis of data for nine GP practices in Greater Manchester with a patient population of 75 421.

**Method:**

Descriptive and comparative analyses were performed for winter and summer periods in 2018–2019. Data were obtained from the North of England Clinical Support Unit (NECSU) via the Rapid Actionable Insight Driving Reform (RAIDR) toolkit, and EMIS Enterprise clinical audit tools.

**Results:**

Accident and emergency (A&E) attendances increased by 4% (*P* = 0.035) during winter with no difference in the number of hospital admissions (*P* = 0.668). The number of problems (defined as separate diagnoses or causes for a GP consultation, for example, chest infection or medication request) seen in general practice increased by 61% (*P*<0.001) in winter compared to summer, as did the number of GP consultations, which was also 61% (*P*<0.001). Respiratory diagnoses saw the greatest seasonal variation accounting for 10% in winter compared with 4% in summer (*P*<0.001). Self-referral accounted for 66% of all A&E attendance and increased by 16% in winter. GP referral accounted for 7% in winter and 6% in summer (*P* = 0.002).

**Conclusion:**

General practice observed a greater seasonal increase in presenting patients compared with secondary care. Any winter pressures strategy should target both respiratory illness and patients who self-refer to A&E. Transferring 50% of self-referrals in Manchester to GP appointments would achieve a £2.3 million cost saving. Increasing provision in primary care requires funding and increased appointments, but more importantly improved patient opportunities to easily access timely advice and assistance.

## How this fits in

It has previously been shown that the number of A&E attendances decrease during the winter, but there has been a lack of research regarding seasonal variation in general practice and the contribution made to A&E attendance by general practice referrals. This study quantifies the difference in seasonal workload, and details the most common diagnoses seen in A&E in winter compared with summer. The findings can help guide GP practices and commissioning bodies in planning for winter pressures.

## Introduction

Each winter the media reports on a lack of hospital beds, overflowing A&E departments, and queues of ambulances. There is an ongoing narrative regarding winter pressures on the NHS with a focus on secondary care and extra funding to assist hospitals and emergency departments during the winter months. The Nuffield Trust’s report on NHS winter pressures highlights a lack of data for primary care, with a consequential focus on secondary care.^1^ The scale of the extra demand has not been well documented and without knowing this it is difficult to make an argument for winter pressures investment in general practice. Where funding is available it is not clear where best to invest it, as there appears to be a lack of evidence as to what works to address additional winter workload in general practice. It is possible that in focusing funding towards secondary care an opportunity is being missed. Directing more towards general practice may reduce winter pressures on both primary and secondary care.

It has been previously shown that A&E attendance falls in winter but emergency admissions mostly caused by respiratory illness rise.^1^ The aims of this study were to identify the presence and scale of seasonal variation in workload on both primary and secondary care. Furthermore, the study sought to characterise the predominant illnesses causing A&E attendance, and establish whether GP referrals contribute to winter workload in secondary care.

## Method

Nine practices from the Manchester Clinical Commissioning Group (CCG) were included in this analysis with a combined list size of 75 421 patients. These practices were selected based on availability, the study did not attempt to investigate differences between practices as data were not available to allow adjustment for confounding factors such as workforce ratios and population demographics; for example, age and deprivation.

Two periods of 3 months were chosen for comparison. Summer was defined as June, July, and August 2018. Winter was defined as December 2018, January, and February 2019.

Secondary care data for each practice were obtained from the Manchester hospitals Secondary Uses Service data through the NECSU, via the RAIDR toolkit.

Data were captured on total patients seen in A&E, mode of arrival, diagnosis, and outcome after attendance. Mode of arrival specifies how patients are referred to A&E; for example, self-referral, GP referral, or referral by another healthcare professional. Outcome after attendance refers to the plan after patients are discharged from A&E; for example, discharged with GP follow-up or admitted to hospital.

Primary care data were obtained using EMIS Enterprise clinical audit tools. Data were captured on the number of problems recorded in a consultation and consultation type; for example, GP surgery consultation, telephone consultation, or home visit. The number of problems are defined as the number of separately recorded diagnoses or issues, raised by either the patient or the doctor, that are discussed in a consultation, for example, chest infection or medication request. Studies have shown that an average of 2.5 problems are discussed in one GP consultation and that GP's record only 81% of these in the medical notes.^2^

Unadjusted risk ratios were calculated for the risk of an event occurring in the winter compared with the summer, along with χ^2^ hypothesis tests.

## Results

Results are expressed as attendances or consultations per 1000 patients to account for different practice list sizes.

### A&E attendance

The total number of A&E attendances were 7521 in winter and 7265 in summer or 100 per 1000 patients in winter, compared with 96 per 1000 in summer (Table 1). There is good evidence for a 4% increase in A&E attendances in winter (*P* = 0.035).

**Table 1.** A&E attendance per 1000 patients. Also shown is the relative risk of A&E attendance in winter compared with summer

	Attendance per 1000 patients, *n*		
Practice	Winter	Summer	RR	*P* value
A	102	96	1.06	0.206
B	102	95	1.07	0.188
C	89	83	1.07	0.065
D	105	105	0.99	0.909
E	122	101	1.20	<0.001
F	65	67	0.96	0.421
G	104	113	0.92	0.225
H	135	132	1.02	0.683
I	125	130	0.96	0.361
Total	100	96	1.04	0.035

RR = relative risk.

There was large variation in A&E attendances between the practices. For example, practice F had 65 attendances per 1000 patients while practice H had 135 per 1000 patients.

#### Diagnosis on A&E attendance

The 10 most common diagnoses on A&E attendance (Table 2) were:

**Table 2.** Most common diagnosis after A&E. Also shown is the relative risk of diagnosis in winter compared with summer

	Attendance per 1000 patients, *n*		
**Diagnosis**	**Winter**	**Summer**	**RR**	***P* value**
No diagnosis	9	11	0.84	<0.001
Nothing abnormal	7	8	0.81	<0.001
Respiratory	10	4	2.31	<0.001
Gastrointestinal	7	7	0.99	0.874
Diagnosis not classifiable	6	5	1.11	0.135
Gynaecological	4	4	0.84	0.028
Ophthalmological	4	3	1.41	<0.001
Dislocation or fracture or joint injury	3	5	0.54	<0.001
Laceration	1	4	0.36	<0.001
Sprain	3	3	1.05	0.615

RR = relative risk.

- ‘No diagnosis’ accounting for 9% of all A&E attendance in winter and 11% in summer.
- ‘Nothing abnormal’ accounting for 7% in winter and 9% in summer.
- ‘Respiratory’ accounting for 10% in winter and 4% in summer.
- ‘Gastrointestinal’ accounting for 7% in both winter and summer.
- ‘Diagnosis not classifiable’ accounting for 6% in winter and 5% in summer.
- ‘Gynaecological’ accounting for 4% in winter and 5% in summer.
- ‘Ophthalmological’ accounting for 5% in winter and 3% in summer.
- ‘Dislocation or fracture or joint injury’ accounting for 3% in winter and 5% in summer.
- ‘Laceration’ accounting for 1% in winter and 4% in summer.
- ‘Sprain’ accounting for 3% in winter and 3% in summer.

The biggest seasonal variation was in respiratory diagnoses with 4 per 1000 patients in summer rising to 10 per 1000 patients in winter (relative risk [RR] = 2.31; *P*<0.001).

There is also good evidence that patients are more likely to present to A&E with an ophthalmological problem in the winter (RR = 1.41; *P*<0.001).

Diagnoses less likely to present to A&E in the winter include ‘no diagnosis’ (RR = 0.84; *P*<0.001), ‘nothing abnormal’ (RR = 0.81; *P*<0.001), ‘dislocation or fracture or joint injury’ (RR = 0.54; *P*<0.001), and ‘laceration’ (RR = 0.36; *P*<0.001).

#### Modes of arrival to A&E

The most common mode of arrival to A&E, in both winter and summer, was self-referral, with 70 per 1000 patients in winter and 60 per 1000 in summer (Table 3). There was strong evidence for an increase in self-referral in winter compared with summer (RR = 1.16; *P*<0.001). Self-referral accounted for 70% of A&E attendance in winter and 63% in summer.

**Table 3.** Most common modes of arrival in A&E. Also shown is the relative risk of referral mode in winter compared with summer

	Self-referral	Referral by another healthcare provider	GP referral
	Attendance per1000 patients, *n*			Attendance per1000 patients, *n*			Attendance per1000 patients, *n*		
Practice	Winter	Summer	RR	*P* value	Winter	Summer	RR	*P* value	Winter	Summer	RR	*P* value
A	71	63	1.13	0.027	18	16	1.14	0.247	10	7	1.42	0.028
B	73	56	1.29	<0.001	19	21	0.93	0.537	6	4	1.31	0.222
C	64	54	1.18	<0.001	13	14	0.96	0.696	5	5	0.97	0.870
D	76	67	1.15	0.048	18	19	0.96	0.785	8	7	1.10	0.663
E	90	65	1.39	<0.001	21	16	1.25	0.062	8	6	1.47	0.052
F	47	45	1.06	0.336	8	9	0.91	0.488	6	5	1.34	0.086
G	60	68	0.89	0.176	9	9	1.00	1.000	6	6	1.00	1.000
H	80	75	1.07	0.400	14	13	1.13	0.521	13	10	1.27	0.235
I	87	77	1.13	0.023	26	30	0.88	0.180	6	6	1.08	0.692
Total	70	60	1.16	<0.001	17	16	1.05	0.252	7	6	1.22	0.002

RR = relative risk.

The second most common mode of arrival in both winter and summer, showing no seasonal difference, was referral by another healthcare provider, with 17 per 1000 patients in winter and 16 per 1000 in summer (*P* = 0.252). This accounted for 17% of all A&E attendance in both winter and summer.

The third most common mode of arrival were GP referrals with seven per 1000 patients in winter and six per 1000 in summer. There was strong evidence for an increase in GP referral in winter compared with summer (RR = 1.22; *P* = 0.002). GP referral accounted for 7% of all A&E attendance in winter and 6% in summer.

There was wide variation in GP referral rate between practices. Practice H had the highest winter referral rate, 13 per 1000 patients, while practice C had the lowest, five per 1000 patients.

#### A&E outcomes

The most common outcome after attendance to A&E was ‘did not require follow-up’ with 65 per 1000 patients in winter and 63 per 1000 in summer (Table 4), showing good evidence for an increase in winter compared with summer (RR = 1.05; *P* = 0.029). Patients that did not require follow-up accounted for 66% of all A&E attendances in winter and 65% in summer.

**Table 4.** Most common A&E outcomes. Also shown is the relative risk of outcome in winter compared with summer

	Did not require follow-up	Admitted to hospital bed	Discharged with GP follow-up
	Attendance per 1000 patients, *n*			Attendance per 1000 patients, *n*			Attendance per 1000 patients, *n*		
Practice	Winter	Summer	RR	*P* value	Winter	Summer	RR	*P* value	Winter	Summer	RR	*P* value
A	70	65	1.08	0.164	9	8	1.04	0.812	8	8	1.07	0.686
B	74	68	1.10	0.101	6	7	0.82	0.327	6	7	0.89	0.560
C	55	53	1.02	0.625	10	10	1.07	0.573	9	6	1.46	0.003
D	73	72	1.01	0.891	9	9	0.94	0.770	8	9	0.91	0.622
E	87	71	1.22	0.001	7	7	1.10	0.626	11	8	1.37	0.071
F	42	43	0.97	0.627	5	4	1.05	0.780	7	6	1.13	0.443
G	51	53	0.96	0.652	25	22	1.12	0.459	15	19	0.79	0.190
H	66	67	0.98	0.773	34	34	1.00	1.000	14	15	0.91	0.599
I	89	89	1.00	0.938	9	13	0.69	0.011	10	11	0.98	0.879
Total	65	63	1.05	0.029	10	11	0.98	0.668	9	9	1.07	0.191

RR = relative risk.

The second most common outcome was ‘admitted to hospital bed’. There was no difference in number of hospital admissions between summer and winter (*P* = 0.668). In total, 10% of A&E attendances were admitted to a hospital bed in winter, 11% in summer.

The third most common outcome was ‘discharged with GP follow-up’, which accounted for 7% of all A&E attendances in both winter and summer.

There was wide variation between practices in hospital admission rate. In winter practice H had the highest rate with 34 per 1000 patients, while practice F had the lowest with 5 per 1000 patients.

### Seasonal variation in general practice patient contact

There were 16 444 more problems seen in general practice in winter compared with summer. This was a 61% seasonal increase (*P*<0.001).

**Figure 1.**
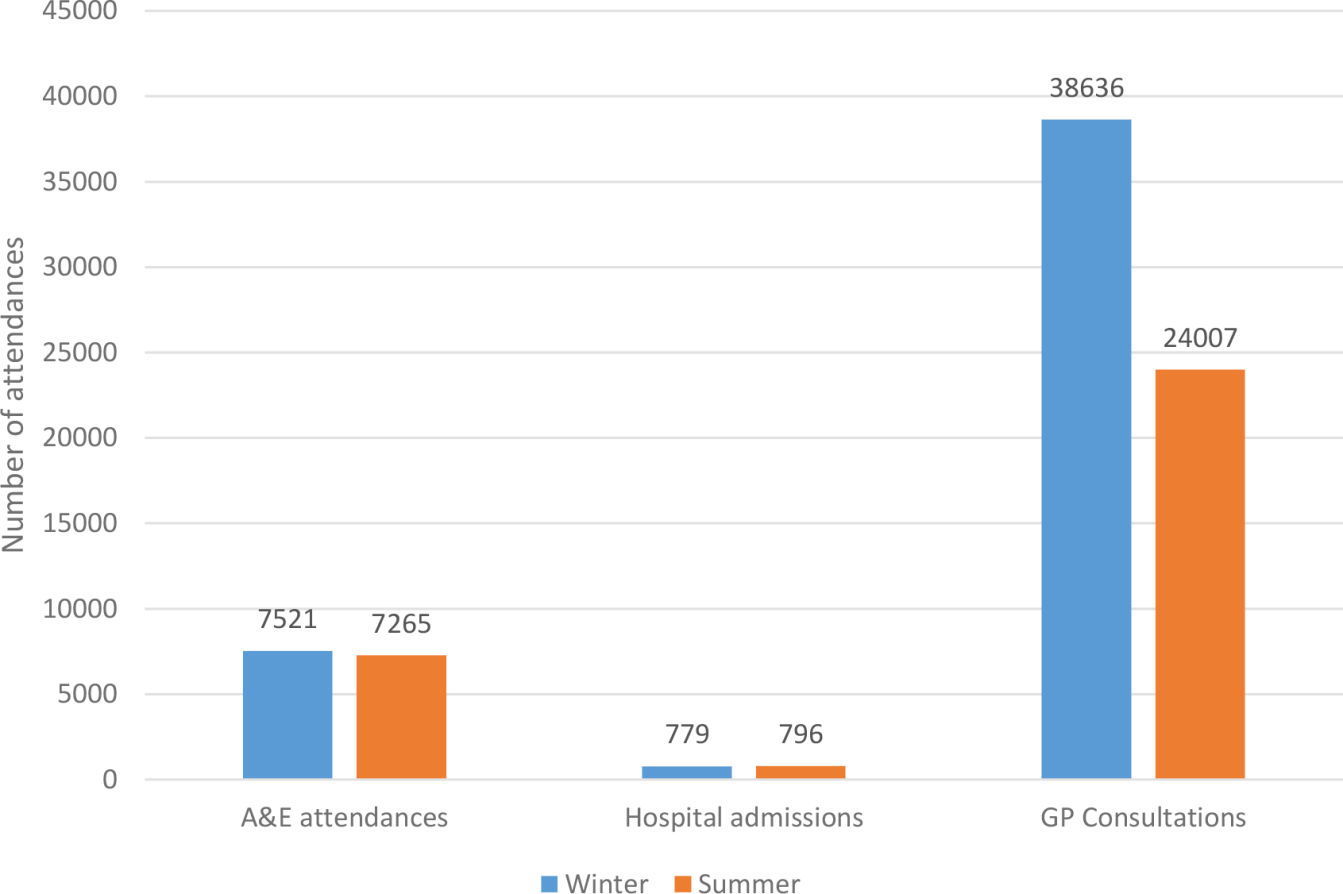
Comparison of seasonal variation in primary and secondary care

There was also a 61% increase in the number of face-to-face GP consultations in winter compared with summer (RR = 1.61; *P*<0.001) (Figure 1). Consultations increased from 318 per 1000 patients in summer to 512 per 1000 in winter (Table 5).

**Table 5.** Comparison of types of GP consultation

	Attendance per 1000 patients, *n*		
Type of consultation	Winter	Summer	RR	*P* value
GP face-to-face consultation	512	318	1.61	<0.001
Telephone consultation	47	26	1.81	<0.001
Home visit	8	3	2.57	<0.001

There is strong evidence that telephone consultations increased from summer to winter from 26 per 1000 patients to 47 per 1000 patients, an 81% increase (RR = 1.81; *P*<0.001).

Home visits also increased from summer to winter from 3 per 1000 patients to 8 per 1000 patients (RR = 2.57; *P*<0.001).

## Discussion

### Summary

Strong evidence for increased workload during the winter months was identified in both general practice and secondary care, with the burden of winter attendance appearing to lean more heavily on general practice.

In the study, secondary care experienced a 4% increase in A&E attendances and no difference in the volume of hospital admissions in winter.

In general practice the study has demonstrated a much greater seasonal variation with a 61% increase in both the number of problems seen and the number of GP face-to-face consultations. There was also an 81% increase in telephone consultations and more than double the number of home visits in winter.

The biggest seasonal variation in diagnosis were respiratory problems, accounting for 4% of the overall A&E attendance in summer and 10% in winter; the risk of attending A&E with a respiratory problem more than doubled in winter.

Wide variations in A&E attendance, GP referral rate, and hospital admission between the practices were identified. It was not the aim of this study to explain variations between individual practices; there may be many reasons for this including workforce ratios, population age, deprivation, and proximity to A&E. Further research into comparing performances between practices is urgently needed; identifying practices with low rates of A&E attendance, after adjusting for confounding factors, might enable their approaches to be replicated.

In the entire 6 months studied, GP referral accounted for only 7% of all A&E attendance. Self-referral accounted for 66% and increased by 16% in winter. In total, 18% of patients were discharged with no diagnosis or nothing abnormal detected, and 65% did not require follow-up. It, therefore, seems logical to expand the role of general practice to manage the bulk of these patients in the community.

### Strengths and limitations

This study benefits from a large dataset, encompassing a population of >75 000 patients. Although selected on availability rather than at random, the practices were from six out of 12 neighbourhoods of Manchester, reflecting a representative sample of practice types and patient populations. The data sources are overall of high quality, routinely used for regional analysis.

One limitation is that significant variation is likely to exist between practitioners in how they record problems. Similarly, in some practices, a consultation may be recorded when patient contact does not occur; for example, when making administrative notes. Seasonal comparisons should not be affected by this but comparisons between practices may be more problematic.

### Comparison with existing literature

This study benefits from direct comparison of general practice data to secondary care data for the same practice population. This has allowed the working relationship between general practice and secondary care to be scrutinised and activity to be directly compared, demonstrating for the first time the clear seasonal difference in workload between general practice and secondary care. It is perhaps unsurprising that GP’s see more patients in winter. Previous research has shown that patients with flu-like illnesses preferentially attend their GP; attendance rates for these illnesses are approximately 10 times higher in daytime primary care than A&E or out-of-hours (OOH) services.^1^

The small increase in A&E attendance and no change in hospital admissions in winter indicates that the well-documented winter increases in A&E waiting times, hospital length of stay, and demand for winter pressures wards cannot be explained simply by an increase in attendance. Other contributing factors include sicker patients requiring longer admissions and a lack of social care beds,^3^ causing long delays in discharge from hospital, with a resultant impact on patient flow in A&E. Once bed occupancy reaches 92%, hospitals become increasingly unlikely to admit patients within the 4-hour A&E target.^1^

The present study findings corroborate what has many times been demonstrated, that respiratory illness increases in the winter. Incidence of respiratory viruses increase in cold weather^3^ putting pressure on NHS services and more importantly, causing severe health consequences. For every 1^°^C drop in temperature below the optimum threshold for different age groups, the risk of death from a respiratory condition increases by approximately 10%.^1^ It follows that any winter pressures strategy should include plans to tackle respiratory illness. Evidence-based measures include optimising long-term condition management, achieving higher levels of influenza and pneumococcal vaccine uptake, smoking cessation, and ensuring patients have warm housing.^4,5^

The main burden of attendance in A&E seems to be from self-referrals and the majority of attendences did not require follow-up, suggesting that many attendances might be better managed by general practice. This could have a beneficial effect on A&E attendance. A 2016 study examining extended access to general practice in Manchester provided evidence that additional primary care appointments, outside of working hours, may reduce attendance at emergency departments.^6^

A crude cost analysis was calculated using the weighted average cost of an A&E attendance in 2019–2020, based on service level activity monitoring files for Manchester University Foundation Trust, and average GP consultation cost as reported by NHS England.^7^ Scaled up to the combined practice population of Manchester, if 50% of the self-referrals to A&E in the winter period of the study were instead managed in general practice, taking into account the 7% GP referral rate to A&E, there would be a cost saving of just over £2.3 million.

There would need to be significant initial investment to support the expansion of general practice to accommodate these extra patients. This is likely to place workforce pressures on an already strained system. However, any barriers to expanding the role of general practice are highly likely to be worth overcoming. In addition to cost saving, the benefit of managing these patients in general practice is much more far reaching and important. There is evidence that continuity of care, encouraged in general practice and not possible in A&E, is associated with reduced mortality.^8^ A study of patients with type 2 diabetes demonstrated that higher levels of empathy from practitioners reduced all-cause mortality.^9^ In addition, GP empathy has been shown to have psychosocial benefits.^10^ Therefore, quite independent of the cost, there are important advantages to any health system for patients to be managed in general practice rather than A&E.

### Implications for practice

The NHS Long Term Plan advises primary care networks (PCNs) to take a proactive approach to managing population health. To incentivise this a ‘shared savings’ scheme is proposed, under which networks will benefit financially from reductions in A&E attendances and hospital admissions.^11^ It is suggested that any winter pressures strategy by a PCN or other organisational body, should target both respiratory illness and patients who self-refer to A&E.

Targeting respiratory illness in the community may reduce A&E attendance and prevent or better manage severe respiratory infections and exacerbations, which would normally result in long hospital admissions.

Targeting the large number of patients who self-refer would increase A&E’s capacity to overcome other factors that prolong waiting times in winter. Existing research can be used to inform initiatives to reduce A&E self-referrals. A systematic review reported that patients’ perceptions of access to and confidence in general practice were key factors in low-urgency A&E attendances.^12^ One study found migrant populations often had no primary care provider and sought A&E care for non-urgent health problems owing to difficulties accessing primary health care.^13^

Targeting self-referrals by increasing provision in primary care would seem sensible. However, as general practice is already managing the vast majority of the NHS’s winter workload, significant new provision is needed.

There could be a combination of approaches. Commissioners could balance winter funding in accordance with the distribution of the workload demonstrated in this study, redirecting more towards general practice. In addition, novel methods enabling general practice to manage more patients with the same resources need to be identified and tested. These are likely to include triage systems and the wider use of allied healthcare practitioners. Successfully improving access to GP services is probably not merely a case of increasing the number of consultations offered to the public, but rather making general practice more accessible. This means making it easy to access timely healthcare advice and assistance for patients from all backgrounds, 24 hours a day, then, only if appropriate, face-to-face assessment.
